# Cumulative effect of simvastatin, l-arginine, and tetrahydrobiopterin on cerebral blood flow and cognitive function in Alzheimer’s disease

**DOI:** 10.1186/s13195-022-01076-7

**Published:** 2022-09-17

**Authors:** Elizabeth Degrush, Mohammed Salman Shazeeb, David Drachman, Zeynep Vardar, Clifford Lindsay, Matthew J. Gounis, Nils Henninger

**Affiliations:** 1 Department of Neurology, University of Massachusetts Chan Medical School, 55 Lake Ave, North, Worcester, MA 01655 USA; 2 Department of Psychiatry, University of Massachusetts Chan Medical School, 55 Lake Ave, North, Worcester, MA 01655 USA; 3 Image Processing and Analysis Core (iPAC), Department of Radiology, University of Massachusetts Chan Medical School, Worcester, MA USA; 4 New England Center for Stroke Research, Department of Radiology, University of Massachusetts Chan Medical School, Worcester, MA USA

**Keywords:** Alzheimer’s disease, Vascular disease, Statin, Endothelial nitric oxide synthase, Dementia, Arterial spin labeling, Perfusion-weighted imaging

## Abstract

**Background and objectives:**

Vascular disease is a known risk factor for Alzheimer’s disease (AD). Endothelial dysfunction has been linked to reduced cerebral blood flow. Endothelial nitric oxide synthase pathway (eNOS) upregulation is known to support endothelial health. This single-center, proof-of-concept study tested whether the use of three medications known to augment the eNOS pathway activity improves cognition and cerebral blood flow (CBF).

**Methods:**

Subjects with mild AD or mild cognitive impairment (MCI) were sequentially treated with the HMG-CoA reductase synthesis inhibitor simvastatin (weeks 0–16), l-arginine (weeks 4–16), and tetrahydrobiopterin (weeks 8–16). The primary outcome of interest was the change in CBF as measured by MRI from baseline to week 16. Secondary outcomes included standard assessments of cognition.

**Results:**

A total of 11 subjects were deemed eligible and enrolled. One subject withdrew from the study after enrollment, leaving 10 subjects for data analysis. There was a significant increase in CBF from baseline to week 8 by ~13% in the limbic and ~15% in the cerebral cortex. Secondary outcomes indicated a modest but significant increase in the MMSE from baseline (24.2±3.2) to week 16 (26.0±2.7). Exploratory analysis indicated that subjects with cognitive improvement (reduction of the ADAS-cog 13) had a significant increase in their respective limbic and cortical CBF.

**Conclusions:**

Treatment of mild AD/MCI subjects with medications shown to augment the eNOS pathway was well tolerated and associated with modestly increased cerebral blood flow and cognitive improvement.

**Trial registration:**

This study is registered in https://www.clinicaltrials.gov; registration identifier: NCT01439555; date of registration submitted to registry: 09/23/2011; date of first subject enrollment: 11/2011.

**Supplementary Information:**

The online version contains supplementary material available at 10.1186/s13195-022-01076-7.

## Background

Alzheimer’s disease (AD) is a degenerative disorder of unknown etiology, characterized by progressive dementia and hallmark pathology at postmortem of amyloid beta plaques and neurofibrillary tangles in the brain [1]. Moreover, several studies have shown that AD patients have decreased cerebral blood flow (CBF) and metabolism and decreased endothelial nitric oxide synthase (eNOS) resulting in a markedly atrophic microvascular endothelium of the brain [2], suggesting that failing endothelial health plays a critical role in the pathogenesis of AD [2–10]. Although these changes may be a consequence of altered brain structure and diminished functional demand, the consistent epidemiologic association of multiple vascular risk factors with the incidence of AD suggests that the vascular changes may represent a causal factor, initiating or contributing to the pathogenesis and development of AD [8–10]. For this reason, therapies known to enhance eNOS and support the structural and functional integrity of the brain’s microvascular endothelium may increase cerebral perfusion and improve cognition in AD [2].

In this respect, drugs acting on the eNOS pathway may be particularly promising because eNOS is critical for microvascular endothelial health [3–5, 11–13]. The primary purpose of this open-label, proof-of-concept, phase II study was to determine whether the combined treatment with drugs known to augment the eNOS pathway could improve CBF. Specifically, we sought to determine whether sequential administration of the 3-hydroxy-3-methylglutaryl coenzyme A (HMG-CoA) reductase synthesis inhibitor simvastatin (which increases eNOS activity) [3], l-arginine (a substrate for nitric oxide in the eNOS pathway [NO]) [4], and the tetrahydrobiopterin (THB; a critical cofactor in the eNOS pathway) [5] could increase CBF as assessed by contrast-enhanced perfusion magnetic resonance imaging (MRI). Prespecified secondary outcomes were the performance of the subjects on the psychometric battery over the 16-week study period to determine whether CBF augmentation was related to cognitive function. Lastly, we conducted exploratory analyses examining global and regional CBF signatures stratified by participants’ cognitive trajectories (improved, unchanged, declined).

## Methods

### Standard protocol approvals, registrations, and subject consents

The trial was conducted at the University of Massachusetts Chan Medical School (UMASS Chan) Neurology dementia clinic in accordance with the principles of Good Clinical Practice guidelines and approved by the UMASS Chan institutional review board according to their ethical standards for human research. Written informed consent was provided by the subjects or their legal representatives. Data were collected and analyzed by the investigators. All the authors approved the manuscript, had full access to the trial data, and vouch for the accuracy and completeness of the data, for the fidelity of the trial to the protocol, and for the reporting of adverse events (except Dr. Drachman who passed away prior to the completion of the data analysis).

### Subjects

Subjects were enrolled in the study between 1/1/2011 and 2/6/2016 and were eligible for enrollment in the study if they were between 55 and 85 years of age and if they had mild AD or mild cognitive impairment (MCI) according to the specifications by the National Institute of Neurological and Communicative Disorders and Stroke (NINCDS) and the Alzheimer’s Disease and Related Disorders Association (ADRDA) Workgroup [14]. Subjects could have been receiving an acetylcholinesterase inhibitor, memantine, or both, provided that they had received a stable dose for at least 3 months prior to study entry. Baseline MRI was performed prior to initiation of any study drugs. Subjects taking statin medication before study entry were allowed study entry after a washout period of at least 8 weeks before the baseline MRI.

Exclusion criteria were known allergy to any of the study drugs; significant psychiatric disorder; history of stroke; current use of any of the test medications (statin, THB, l-arginine); active malignancy; renal insufficiency (elevated creatinine above 1.3 mg/dL); other serious diseases including coronary insufficiency or congestive heart failure (ICD-9 criteria); known carotid stenosis, active peptic ulcer; urinary tract infection; and inability to come to the study site for follow-up.

### Trial design

This was a single-center, single-arm, prospective proof-of-concept study that assessed adverse events and effects on CBF (primary aim) and cognition (secondary aim) of sequential treatment with simvastatin, l-arginine, and THB in subjects with AD or MCI. We chose these drugs based on their known interaction with the eNOS pathway in a potentially synergistic manner:

1. The HMG-CoA reductase synthesis inhibitor simvastatin has been shown to upregulate eNOS expression [15] as well as inhibit the Rho-kinase (ROCK) pathway, which leads to rapid phosphorylation and activation of eNOS via the phosphatidylinositol-3 kinase (PI3K)/protein kinase B (PKB/Akt) [16, 17]. This results in enhanced eNOS activity, which promotes nitric oxide (NO) production and subsequently improves cerebral perfusion [18–21].
2. l-Arginine, a semi-essential amino acid, is the substrate used by eNOS to produce NO in the vascular endothelium [22, 23]. Following simvastatin-induced eNOS upregulation, l-arginine amplifies and sustains cerebral hyperemia [21].
3. THB is an essential cofactor of the eNOS. Low bioavailability of THB leads to uncoupling of eNOS favoring the production of the superoxide oxide over NO. Conversely, supplementation of THB improves endothelium-dependent vasodilation and treatment with simvastatin elevates endothelial THB through inhibition of the ROCK pathway in vitro.

After informed consent, eligible subjects underwent formal history taking, physical and neurologic examination, psychometric assessment, blood work, and brain MRI (Table 1). Seven subjects also underwent lumbar puncture, and 1 subject underwent nuclear imaging to rule out other possible causes of dementia as part of their routine care. Although we included MCI as well as AD in our inclusion criteria, only 1 subject was diagnosed with MCI at the start of the study. This subject transitioned to AD early in the study according to the NINCDS and ADRDA Workgroup definition. Therefore, no attempt was undertaken to stratify the analyses in this study according to the diagnosis of MCI versus AD due to our small study size and lack of power for this type of analysis.

**Table 1:** Baseline demographics

Baseline characteristics	Included subjects (*n*=10)
Age in years	67 (7)
Gender	60% women, 40% men
Hypertension	40%
Coronary artery disease	0%
Hyperlipidemia	10%
Baseline cognitive assessment	
Mini-Mental State Examination	24 (3)
Cognitive Assessment Screening Test	29.6 (5)
Clinical Dementia Rating scale	1 (0.47)
ADAS-cog 13	31 (7)
Serology	
C-reactive protein (mg/L)	1.4 (1.8)
Low-density lipoprotein (mg/dL)	137.2 (26.7)
High-density lipoprotein (mg/dL)	58.2 (19.2)
Total cholesterol (mg/dL)	223.9 (32.3)
Triglycerides (mg/dL)	133.1 (66.9)
Creatine phosphokinase (U/L)	69 (36.86)
Thyroid-stimulating hormone (mU/L)	2.92 (2.33)
Vitamin B12 (pg/mL)	506 (359)
Erythrocyte sedimentation rate (mm/h)	8.8 (5.9)
International normalized ratio	1.0 (0.1)
Medication use	
Donepezil or rivastigmine	80%
Memantine	40%
Psychotropic use	70%
Anticholinergic drug use^a^	30%
Apolipoprotein E (APOE) genotyping (*n*=9)	
APOE-3,3	1 (11%)
APOE-3,4	5 (56%)
APOE-4,4	3 (33%)
Cerebral spinal fluid phosphorylated tau (*n*=7)	
High (>68 pg/mL)	3(43%)
Intermediate (55–67 pg/mL)	1 (14%)
Normal (<54 pg/mL)	3 (43%)

ADAS-cog 13 indicates Alzheimer’s Disease Assessment Scale-cognitive 13. Data are shown as mean±sd or %. ^a^Most commonly diphenhydramine

**Fig. 1:**
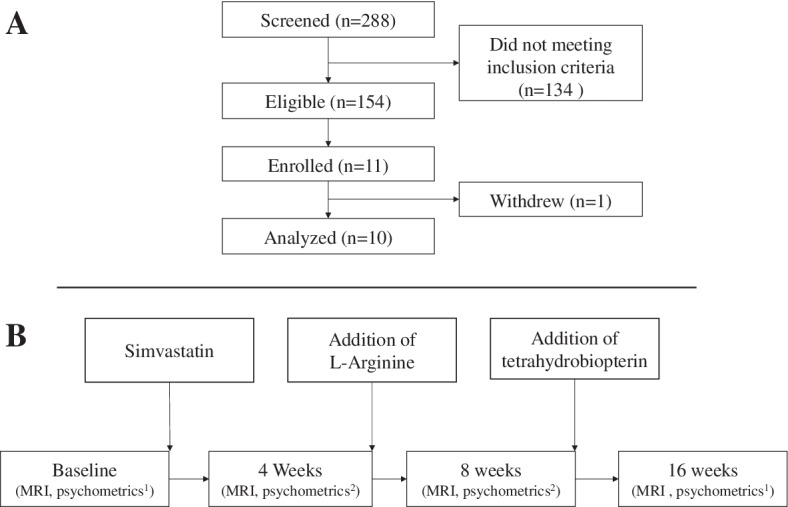
Study design and timeline. **A** Study flowchart. **B** Study timeline depicting the timing of MRI and specific psychometric analysis relative to treatment initiation. ^1^Included Alzheimer’s Disease Assessment Scale-Cognitive 13 (ADAS-cog 13), Clinical Dementia Rating Scale (CDR), Cognitive Assessment Screening Test (CAST), and Mini-Mental State Exam (MMSE). ^2^Included Clinician Interview-Based Impression of Change plus caregiver input (CIBIC-plus) and MMSE. ^3^Included ADAS-cog 13, CDR, CIBIC-plus, and MMSE

After baseline assessment, subjects were sequentially treated once daily with the three study drugs. From weeks 0 to 16, subjects received oral simvastatin at a dose of 40 mg at bedtime; from weeks 4 to 16, subjects received oral l-arginine at a dose of 2 g three times a day and at bedtime; from weeks 8 to 16, subjects received oral THB at a dose of 20 mg/kg once a day. The study timeline is shown in Fig. 1. After completion of the 16-week study, subjects had the choice of continuing to take the three study medications or to taper and discontinue them over 8-day periods for each drug (THB first, l-arginine second, simvastatin third).

### Imaging protocol and image analysis

Dynamic susceptibility contrast (DSC)-MRI was performed on a Philips Achieva 3.0T/60-cm bore magnet (Philips Healthcare, Andover, MA, USA) scanner with gadolinium (0.1 mmol/kg) for all subjects to assess brain perfusion at 4 time points after initiation of the treatment regimen: baseline, 4 weeks, 8 weeks, and 16 weeks. The imaging protocol included DSC-MRI (TR/TE = 1700/40ms, FA = 75°, 100 dynamics, matrix = 128×128) and T1-MPRAGE (TR/TE = 7/3ms, FA = 8°, matrix = 256×256). In an exploratory post hoc analysis, the MRI perfusion parameters were analyzed and stratified by the degree of cognitive change in subjects as assessed on the ADAS-cog 13.

Volumetric brain analysis was performed at baseline using BrainSuite software [24]. Image analysis was performed using ImageJ (National Institutes of Health, Bethesda, MD, USA) and DSC-MRI toolbox in MATLAB (Mathworks, Natick, MA, USA) with semi-automated arterial input function selection and deconvolution algorithms [25, 26]. CBF, cerebral blood volume (CBV), and mean transit time (MTT) maps were generated for each subject at each time point. To ensure an objective comparison of CBF maps between time points and across all subjects, a relative CBF (rCBF) map was calculated by normalizing the CBF maps relative to the whole brain CBF. Similarly, the CBV and MTT maps were normalized to generate relative CBV (rCBV) and relative MTT (rMTT) maps. CBF analyses were focused on five regions of interest (ROIs, eFigure 1 in Supplement) that comprised the limbic system (hippocampus plus amygdala) and three cortical regions (middle temporal, middle frontal, and inferior parietal lobes). These regions were chosen because prior studies demonstrated reduced perfusion in these areas among subjects with MCI and AD [27, 28]. ROIs were manually placed on perfusion MRI images by a radiologist (Z.V.). Finally, whole brain white matter (WM), gray matter (GM), cerebrospinal fluid (CSF) volumes as well as regional volumes of the limbic system, and select cortical regions were calculated for all the subjects.

### Psychometrics

Cognitive function was evaluated using a battery of psychometric measures including the Mini-Mental State Exam (MMSE), a 30-point scale of cognitive function where higher scores indicate better cognition [29]; the Cognitive Assessment Screening Test (CAST), a 40-point self-administered cognitive screen assessing memory, general intellect, visuospatial functioning, and mathematics where higher scores indicate better cognition [30]; the Clinical Dementia Rating Scale (CDR/sum of boxes), a semi-structured interview of the subject and their primary caregiver to rate impairment in six categories on a 0–3-point scale including memory, orientation, judgment, problem solving, community affairs, home and hobbies, and personal care, with higher scores showing more impairment [31]; and the Alzheimer’s Disease Assessment Scale-Cognitive (ADAS-cog) 13 [32, 33], to assess the severity of cognitive impairment in multiple cognitive domains, with higher scores showing more impairment. We used the Clinician Interview-Based Impression of Change plus caregiver input (CIBIC-plus) [34] to gauge a global impression of change from the caregiver, the subject, and the physician. To reduce variability, each subject was tested by the same trained research assistant.

### Outcomes

The primary predetermined outcome of interest was the change in CBF from baseline to 16 weeks as measured by MRI.

There were five secondary predetermined outcomes: the change from baseline to week 16 in the MMSE, CDR, and ADAS-cog 13 and the change in CIBIC-plus scores between 4 and 16 weeks.

To gain deeper insight into the possible link between CBF augmentation and cognitive outcome measures in the entire cohort, we conducted exploratory post hoc analyses stratified according to the cognitive trajectories as assessed by the ADAS-cog 13. For this analysis, we chose the ADAS-cog 13 (scores range from 0 to 85, with higher scores indicating worse cognition) because it was specifically designed to assess the efficacy of treatments based on cognitive performance for AD patients [33, 35], used as a primary outcome of the landmark donepezil studies, and shown to be sensitive to short-term cognitive changes [36]. We stratified included subjects into three groups (eTable 1 in Supplement): improvement (decline in ADAS-cog 13 by at least one point between baseline and 16 weeks: group 1), stable (16-week ADAS-cog 13 remained within a half point from baseline, group 2), and deterioration (increase in ADAS-cog 13 by at least one point from baseline to 16 weeks: group 3).

### Statistical analysis

Unless otherwise stated, continuous variables are reported as mean ± standard deviation or as median (interquartile range [IQR]). Normality of data was examined using the Shapiro–Wilk test. One-way repeated measures analysis of variance (ANOVA) tests were used to analyze the rCBF and psychometric data for statistical significance over time. rCBF differences between the three subject groups were examined by performing two-sample *t*-tests using the SPM12 software (Statistical Parametric Mapping version 12, Wellcome Department of Imaging Neuroscience, University College London). For the rCBF, rCBV, and rMTT values across the 4 time points for the 3 subgroups, ANOVA for mixed models was used to determine whether there was a significant change in the perfusion parameters across time and between the groups for the limbic system and cerebral cortex. Two-sided significance tests were used throughout and unless stated otherwise, a two-sided *P*<0.05 was considered statistically significant. All statistical analyses were performed using IBM® SPSS® Statistics Version 26 (IBM®-Armonk, NY) and GraphPad Prism (V9.0.2 for Windows, GraphPad Software, La Jolla, CA).

### Protocol and statistical analysis plan

The study protocol and statistical analysis plan were published [37].

## Results

Between January 1, 2011, and February 6, 2016, a total of 288 subjects were screened during a routine appointment in the UMASS Chan Neurology dementia clinic for study eligibility. Of these, 154 were diagnosed utilizing the ICD 9 diagnostic criteria with AD, MCI, or dementia of all types. These subjects were screened for study participation. A total of 11 subjects were deemed eligible and enrolled. One subject withdrew from the study after enrollment, leaving 10 subjects in the study for data analysis. The Consolidated Standards of Reporting Trials (CONSORT) diagram for subject flow is depicted in Fig. 1. Although there were no limitations placed on race, all included subjects were Caucasian. Table 1 summarizes the subjects’ baseline characteristics.

### Primary outcome

**Fig. 2:**
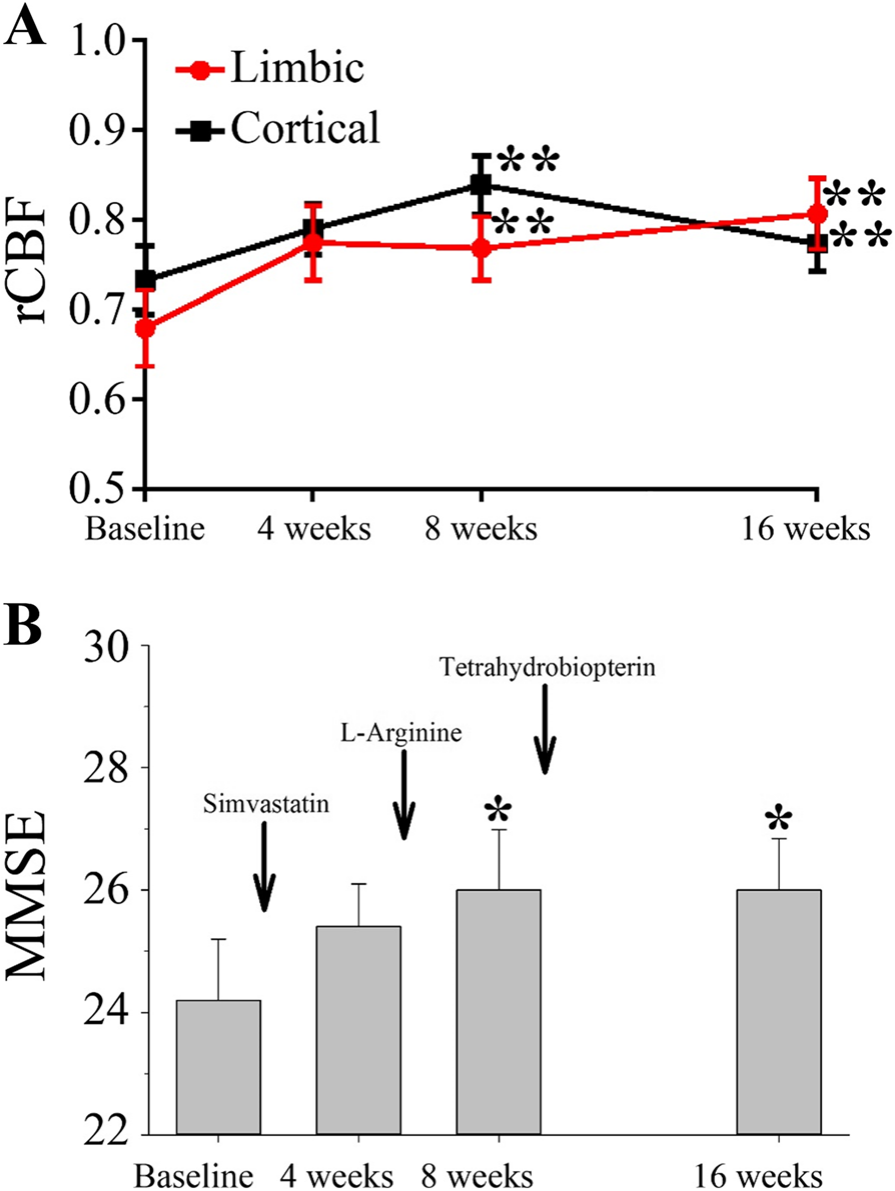
Time-course of relative cerebral blood flow (rCBF) and Mini-Mental State Exam (MMSE) in all subjects. **A** After initiation of treatment, rCBF values significantly increased by weeks 8 and 16 in both the limbic system (red) and the selected cortical areas (black) when compared to the respective baseline values. **B** A modest increase was seen in the MMSE scores from baseline to 8 and 16 weeks. Data are mean ± standard error mean (SEM).*P<0.05, **P<0.01

Comparison of the rCBF across time points for all subjects showed no significant differences in global blood flow (not shown; repeated measures ANOVA using SPM12). However, with a more sensitive ROI analysis, there was a significant difference in the rCBF across time points (*P*=0.001) without a region (limbic versus cortex) and time interaction. Specifically, after initiation of treatment, rCBF values significantly increased by weeks 8 and 16 in both the limbic system (~13% in week 8 and ~19% in week 16) and the selected cortical areas (~15% in week 8 and ~6% in week 16) when compared to the respective baseline values (Fig. 2A).

### Secondary outcome

We observed a modest but significant increase in the mean MMSE scores from baseline (24.2±3.2) to 8 weeks (26.0±3.1) and 16 weeks (26.0±2.7), respectively (*P*<0.05, each) (Fig. 2B). There was no significant difference between baseline and 16-week psychometric scores as assessed by the CDR (*P*>0.99), ADAS-cog 11 (*P*=0.87), and ADAS-cog 13 (*P*=0.43), respectively. There was no difference in the CIBIC-plus scores between 4 and 16 weeks (*P*=0.19). eTable 2 in the Supplement summarizes the data for the psychometric assessments.

### Exploratory post hoc analyses

We identified 3 subjects with cognitive improvement (ADAS-cog 13 declined by 3.6 points by 16 weeks), 3 subjects without substantial change in their ADAS-cog 13 (increase by 0.1 points at 16 weeks), and 4 subjects with cognitive worsening (ADAS-cog 13 increased by 5.8 points by 16 weeks) (eTable 1 in Supplement).

**Fig. 3:**
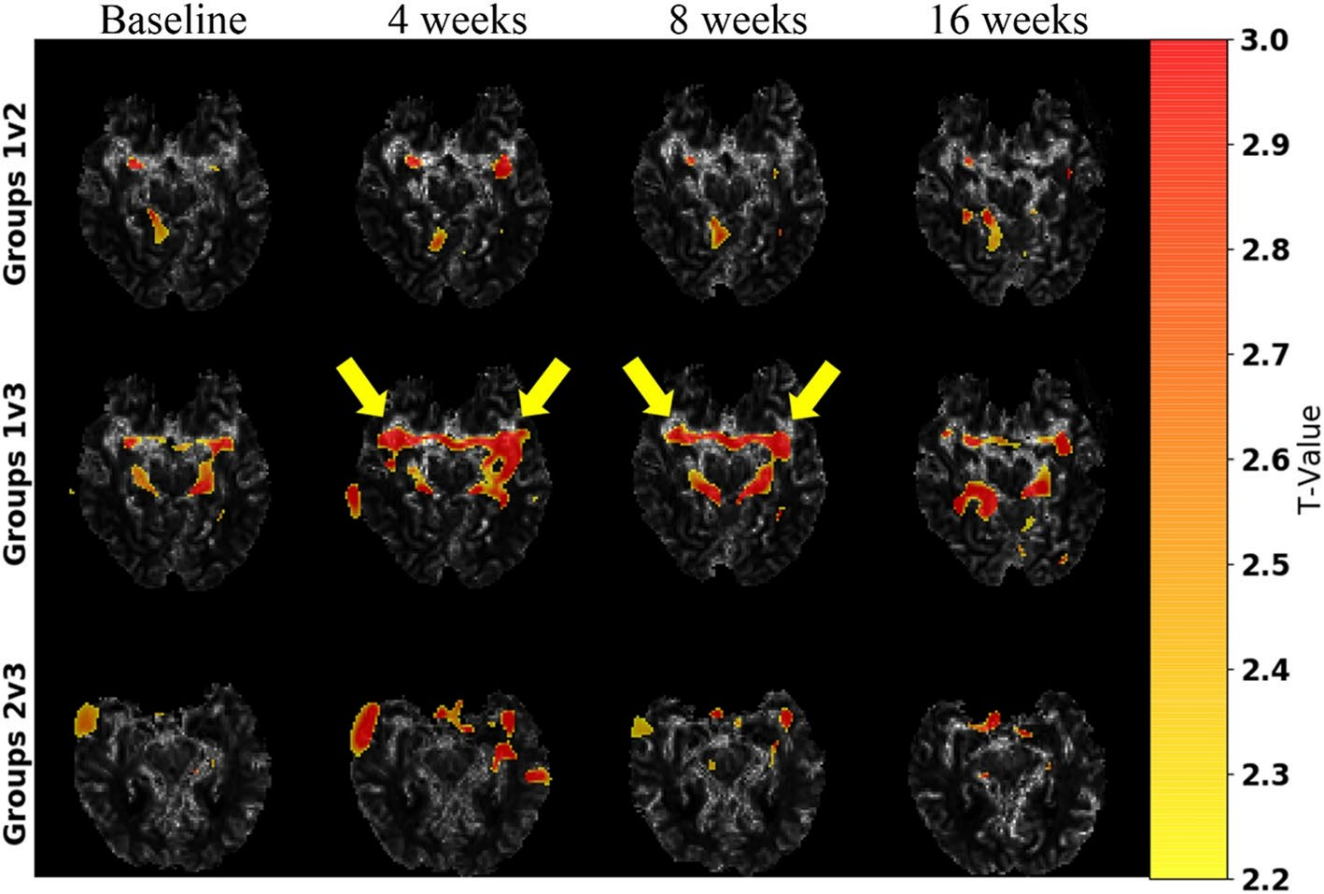
Statistical parametric mapping (SPM) analysis of subject group relative cerebral blood flow (rCBF). *T*-value maps, produced from two-sample *t*-tests using SPM12, were overlaid on a single-subject rCBF map. Shown are axial slices at the level of the circle of Willis and *T*-value maps comparing all subject groups across time points. Regional differences are shown for each group comparison, where higher *T*-values (warmer colors) indicate greater differences. Group 1 (improved cognition) and group 3 (worsened cognition) show the greatest differences in rCBF (uncorrected *P*<.01) particularly at the 4-week and 8-week time points in the bilateral middle cerebral arteries (arrows)

**Fig. 4:**
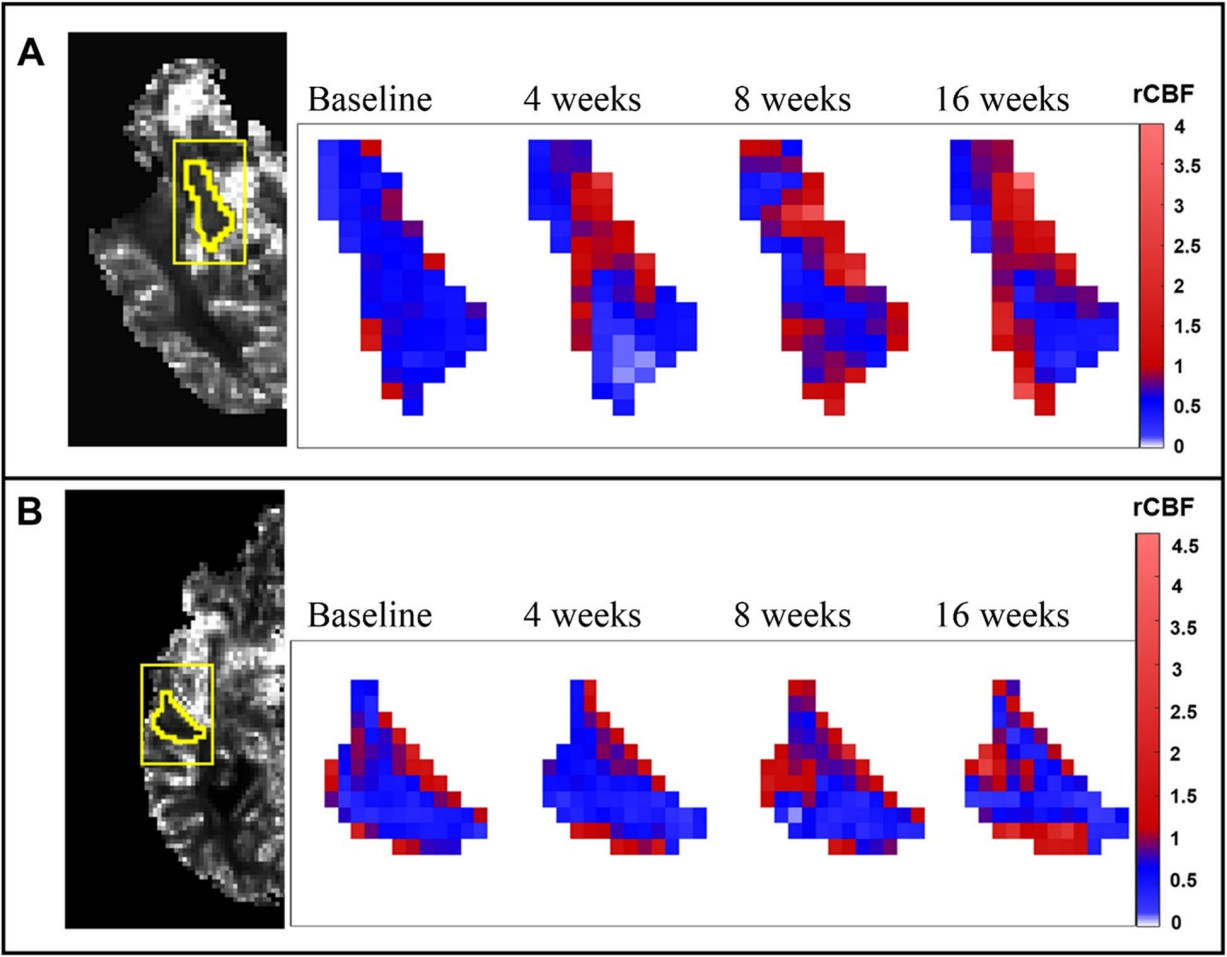
Temporal evolution of cerebral blood flow (CBF) in a group 1 subject. Representative CBF maps showing an increase in relative CBF (rCBF) in the **A** right hippocampus and **B** right middle temporal lobe of subject 1 from group 1 over the course of the study

**Fig. 5:**
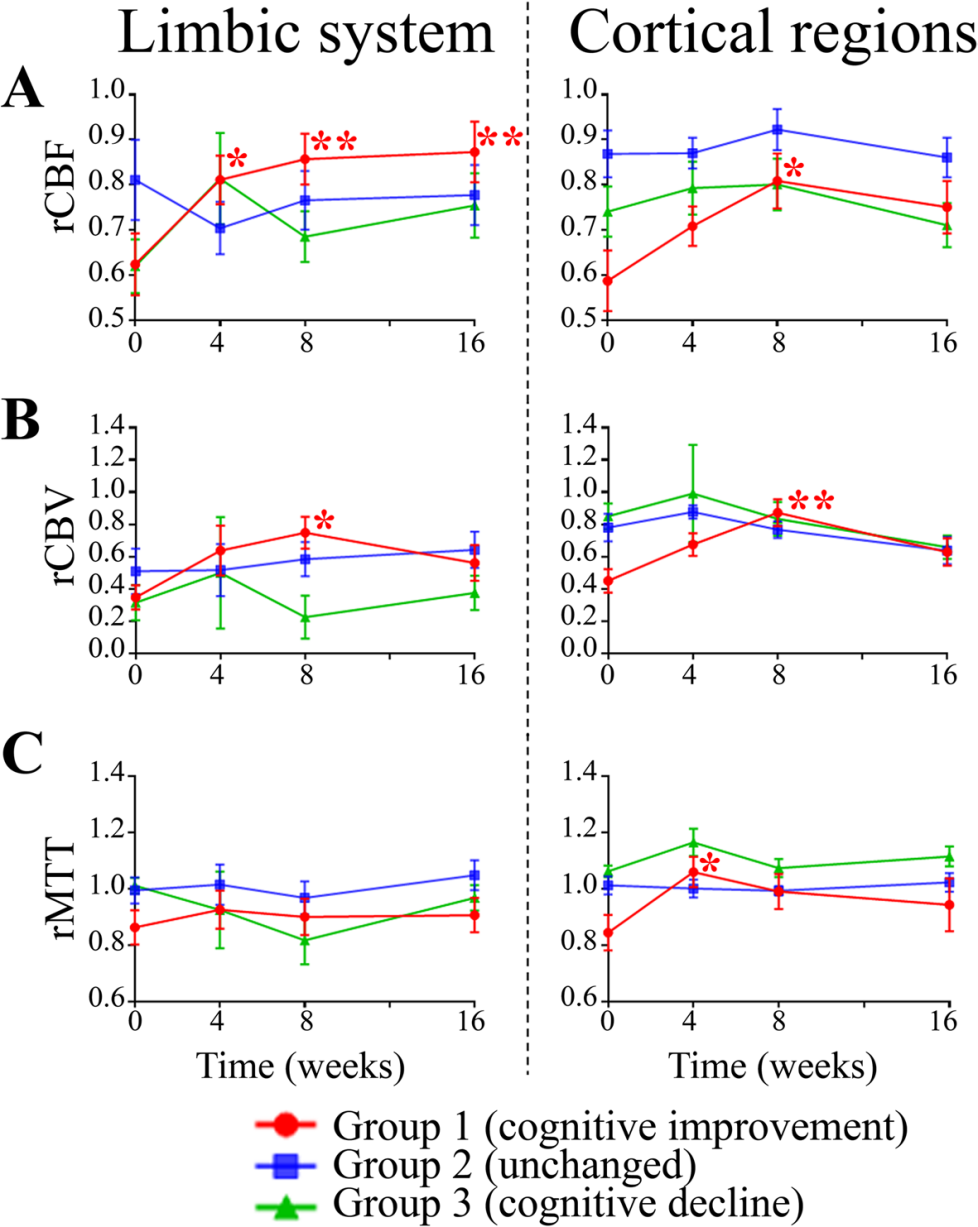
Temporal evolution of assessed cerebral perfusion measures stratified by limbic versus cortical regions of interest. Cumulative summary of DSC-MRI perfusion parameter analysis is shown for **A** relative cerebral blood flow (rCBF), **B** relative cerebral blood volume (rCBV), and **C** relative mean transit time (rMTT). There was a significant increase (mixed ANOVA effect) in limbic rCBF (4–16 weeks), cortical rCBF (8 weeks), limbic and cortical rCBV (8 weeks), and cortical rMTT (4 weeks) relative to baseline in subjects with cognitive improvement (group 1). There were no significant differences in the cerebral perfusion measures in subjects with stable (group 2) and worsened (group 3) cognition as assessed in the limbic and cortical regions. Data are mean ± standard error mean (SEM). *P<0.05, **P<0.01 versus baseline

Volumetric analysis across the different subject groups did not indicate any significant differences in the WM, GM, CSF, limbic system, and cortical region structures at baseline (eFigure 1 in Supplement). Two-sample *t*-tests (SPM12) demonstrated greater differences in blood flow in group 1 (cognitive improvement) as compared to group 3 (cognitive decline) in the middle cerebral artery bilaterally over the course of the study (Fig. 3). Differences were greatest between baseline and 4 weeks (uncorrected *P*<0.0095, higher *T*-values) with attenuated further increases afterwards. Additionally, group-wise comparisons of rCBF (within group subjects) with time interaction were performed using a repeated measures ANOVA (SPM12) for each group individually. This analysis showed no significant differences between the different time points for each group. Conversely, ROI analysis on the rCBF maps showed a significant signal increase in the limbic system (at 4, 8, and 16 weeks) and examined cortical regions (at 8 weeks) in the cognitively improved cohort (group 1) relative to baseline indicating overall increased blood flow (Figs. 4 and 5). There was no significant change in rCBF over time in any of the examined ROIs in subjects with stable (group 2) and worsening (group 3) cognition relative to baseline. Similar to the rCBF, rCBV significantly increased relative to baseline for the cognitively improved group in the limbic system (at 8 weeks) and examined cortical regions (at 8 weeks), without significant change over time in the other two groups. The rMTT only showed a significant increase from baseline to 4 weeks in the examined cortical regions for the cognitively improved group while no significant changes were observed in the other two groups.

## Discussion

Substantial evidence implicates microvascular dysfunction in cerebral hypoperfusion and AD pathogenesis suggesting that pharmacological targeting of pathways that are critical for endothelial health could translate to better cerebral perfusion and, ultimately, improve cognitive outcomes [3–5, 9, 11–13, 38]. The regulation of CBF is in part controlled by the cerebral endothelium via multiple modes such as chemical control of vascular tone, heterotypic and homotypic cell-cell interactions, second messenger signaling, and cellular response to physical forces and inflammatory mediators [39]. Indeed, prior studies suggested that treatment with statins, l-arginine, and THB increased CBF via augmentation of the eNOS system in a number of disease states [40, 41]. Moreover, statin therapy was associated with significant CBF augmentation in a small pilot study of asymptomatic adults at high risk for AD [38]. Yet, it is uncertain whether CBF augmentation relates to improved cognition in those with AD/MCI. The primary goal of this open-label, single-center, prospective study was to determine whether sequential treatment of subjects with AD/MCI with medications shown to augment the eNOS pathway related to CBF improvement.

Indeed, with respect to the predetermined primary outcome of interest, we found a significant increase in the CBF after treatment initiation, specifically within the limbic system (hippocampus and amygdala) as well as the cerebral cortex, which are regions known to have lower CBF in subjects with AD/MCI [27, 28, 42]. Potential reasons for the region-specific response may include differences in baseline metabolism, compensatory mechanisms, degree of cerebral atrophy, and vascular density and angiogenesis [42–45]. For example, postmortem examinations of AD brains indicated that the hippocampus was the only brain region exhibiting both angiogenesis and increased vascular density [44], which could explain the greater response to therapy as compared to other brain regions. Nevertheless, while these observations provide proof-of-concept that medications acting on the eNOS pathway could improve cerebral perfusion status, the specific underlying mechanisms remain to be elucidated [46–50].

Notably, significant betterment of CBF was observed only at the 8-week time point, after subjects had been receiving high-dose simvastatin for 8 weeks and l-arginine for 4 weeks. Conversely, no additional CBF increase was observed after the addition of THB. Since the treatment sequence was not randomly allocated to subjects, it is presently uncertain to what extent the increase in CBF was due to treatment with simvastatin versus l-arginine. Likewise, it is presently unknown whether the lack of further CBF increase with THB was due to a “ceiling effect” (i.e., maximal pharmacological augmentation of the eNOS pathway) or due to the overall lack of efficacy of THB. Given the relatively short study duration, it remains to be shown whether pharmacological intervention results in long-term augmentation of the CBF. Lastly, though suggestive, our study cannot establish causation between the noted improvement in CBF and cognitive function. Thus, our findings should be considered hypothesis generating. Larger studies with random allocation of the treatment sequence and a longer observation period are required to confirm our findings and clarify these important questions. Nevertheless, support for the hypothesis that augmentation of the baseline (resting) CBF could promote cognitive function stems from pre-clinical observations showing that treatment with simvastatin restored neurovascular coupling and improved spatial memory in a mouse model of AD [51].

With respect to our predefined secondary outcomes, we observed a significant, though modest, increase in the MMSE scores from baseline to weeks 8 and 16, which coincided with the time of significant CBF increase. Although the MMSE is a well-known and often used tool in the diagnosis and monitoring of dementia such as AD [29], it has been shown to be insensitive to changes in mild disease and may be insensitive for detecting changes over short periods of time such as in our study [52]. Due to the overall short duration of the observation period, the absence of substantial changes in cognitive measure may not be too surprising and longer observation periods will be required to better understand the potential impact of the chosen medication regimen on cognitive trajectories. It is also possible that the increase in MMSE was influenced by practice effects rather than due to changes in CBF. In this respect, inclusion of sham-treated controls with similar comorbidities could have helped to further contextualize our results and assuage concerns that changes in the secondary outcomes could have been biased by “practice effects.”

Nevertheless, our additional exploratory analyses that stratified subjects according to the change in the ADAS-cog 13 indicated that improvement was positively correlated with the CBF. The ADAS-cog 13 was used in the landmark donepezil studies and showed significant changes in scores at testing intervals as short as 6 weeks, which falls into our study timeline [53]. Importantly, the mean change in ADAS-cog 13 scores in our study was greater than 3, consistent with a clinically meaningful improvement [54]. In addition, several studies done in a larger population cohort found a significant association between higher CBF and improved cognitive function [55, 56]. Together, our observations support the hypothesis that treatment with medications known to act on the eNOS pathway may relate to cognitive improvement through CBF augmentation. Nevertheless, though an exciting possibility, these data should be interpreted with caution given the small subgroup size, absent sham-treated controls, and exploratory nature of the analyses.

Strengths of this proof-of-concept study include prospective determination of predefined outcome parameters including global and regional CBF measurements as well as multimodal assessment of cognitive domains with standardized and well-established psychometrics. Limitations include the small sample size and relatively short observation period, which reduced the power of our analyses. The open-label and unblinded study design, lack of a control group, and absent inclusion of non-whites may have introduced bias. Moreover, drug doses were chosen pragmatically but may not represent the optimal effective dose. Finally, the allocation order of the study drugs was not randomized precluding assessment of the relative contribution of each drug to the study outcomes.

## Conclusions

Treatment of mild AD/MCI subjects with a novel combination of three FDA-approved medications known to augment the eNOS pathway was well tolerated and associated with improvements of CBF and cognitive markers over the 16-week study period. This data may serve as a foundation for future randomized clinical trials to confirm these findings.

## Supplementary Information

**Additional file 1: eTable 1.** Psychometric assessments. **eTable 2.** Individual ADAS-cog 13 scores and group assignment. **eFigure 1.** Regions of interest (ROIs) for cerebral perfusion assessment and brain volumetric measurements.

## Data Availability

Data can be made available on reasonable request by a qualified researcher.

## Abbreviations

AD: Alzheimer’s disease
eNOS: Endothelial nitric oxide synthase
HMG-CoA: 3-Hydroxy-3-methylglutaryl coenzyme A
THB: Tetrahydrobiopterin
CBF: Cerebral blood flow
rCBF: Relative cerebral blood flow
MCI: Mild cognitive impairment
MRI: Magnetic resonance imaging
NINCDS: National Institute of Neurological and Communicative Disorders and Stroke
ADRDA: Alzheimer’s Disease and Related Disorders Association
ROCK: Rho-kinase
PI3K: Phosphatidylinositol-3 kinase
PKB: Protein kinase B
NO: Nitric oxide
DSC: Dynamic susceptibility contrast
CBV: Cerebral blood volume
rCBV: Relative cerebral blood volume
MTT: Mean transit time
rMTT: Relative mean transit time
ROI: Region of interest
WM: White matter
GM: Gray matter
CSF: Cerebrospinal fluid
MMSE: Mini-Mental State Exam
CAST: Cognitive Assessment Screening Test
ADAS-cog: Alzheimer’s Disease Assessment Scale-cognitive
CIBIC: Clinician Interview-Based Impression of Change
CDR: Clinical Dementia Rating
IQR: Interquartile range
ANOVA: Analysis of variance
SPM: Statistical parametric mapping
CONSORT: Consolidated Standards of Reporting Trials
