# Supplementary material for: Cumulative effect of simvastatin, l-arginine, and tetrahydrobiopterin on cerebral blood flow and cognitive function in Alzheimer’s disease
Source: Alzheimers Res Ther. 2022 Sep 17;14:134. doi: 10.1186/s13195-022-01076-7 (PMC9482313; doi:10.1186/s13195-022-01076-7)
Supplement: Supplementary file 1 — Additional file 1: eTable 1. Psychometric assessments. eTable 2. Individual ADAS-cog 13 scores and group assignment. eFigure 1. Regions of interest (ROIs) for cerebral perfusion assessment and brain volumetric measurements. [file 13195_2022_1076_MOESM1_ESM.docx]

## **Supplementary Online Content**

Degrush E, Shazeeb MS, Drachman D, et al. Cumulative effects of simvastatin, L-arginine, and tetrahydrobiopterin on cerebral blood flow and cognitive function in Alzheimer’s disease

eTable 1. Psychometric assessments

eTable 2. Individual ADAS-cog 13 scores and group assignment

eFigure 1. Regions of interest (ROIs) for cerebral perfusion assessment and brain volumetric measurements.

## **eTable 1. Individual ADAS-cog 13 scores and group assignment**

| Patient ID | Baseline | 16-week | Delta | Delta by group | |
| --- | --- | --- | --- | --- | --- |
| Subject 1 | 24.0 | 22.6 | -1.4 | -3.6±2.4 | Group 1 |
| Subject 2 | 35.6 | 29.3 | -6.3 |  |  |
| Subject 3 | 33.3 | 30.3 | -3.0 |  |  |
| Subject 4 | 30.0 | 30.0 | 0 | +0.1±0.1 | Group 2 |
| Subject 5 | 24.0 | 24.0 | 0 |  |  |
| Subject 6 | 32.0 | 32.3 | +0.3 |  |  |
| Subject 7 | 32.0 | 34.0 | +2 | +5.8±2.0 | Group 3 |
| Subject 8 | 20.0 | 27.0 | +7 |  |  |
| Subject 9 | 43.3 | 54.0 | +10.7 |  |  |
| Subject 10 | 36.0 | 39.3 | +3.3 |  |  |

Psychometric assessment of study participants is shown using Alzheimer’s Disease Assessment Scale-Cognitive 13 (ADAS-cog 13) scores. Data are mean ± standard error mean (SEM).

## **eTable 2. Psychometric assessments**

**Baseline 4 weeks 8 weeks 16 weeks**

ADAS-cog 13 31.0±6.9 - - 32.3±9.0

CAST 31.4±5.5 - - -

CDR 0.8 (0.5-1.0) - - 0.8 (0.5-1.0)

CIBIC plus - -0.1±0.3 0.6±0.8 0.1±1.3

MMSE 24.2±3.2 25.4±2.2 26.0±3.1 26.0±2.7

Data are mean±sd or median (25^th^ - 75^th^ percentile). Dashes indicate that this test was not performed at this time-point. ADAS-cog 13 indicates Alzheimer’s Disease Assessment Scale-Cognitive 13; CAST, Cognitive Assessment Screening Test; CDR, Clinical Dementia Rating scale; CIBIC-plus, Clinician Interview Based Impression of Change plus caregiver input; MMSE, Mini Mental State Examination.

**eFigure 1. Regions of interest (ROIs) for cerebral perfusion assessment and brain volumetric measurements.**


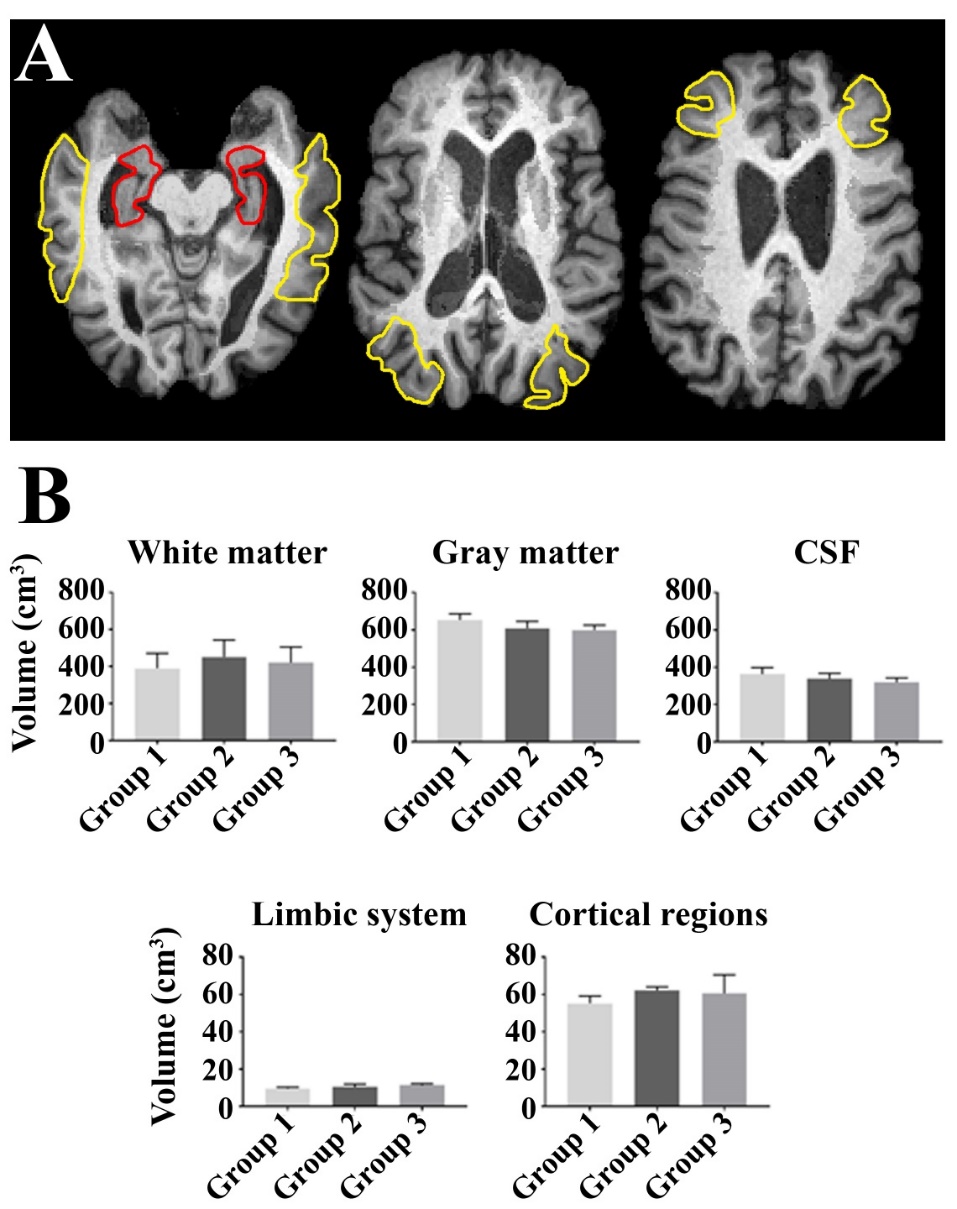


(**A**) Depiction of the ROIs chosen for the assessment of cerebral perfusion measures including the limbic system (red) and cortical areas (yellow). (**B**) At baseline, there was no significant difference in brain volumetrics between Subjects with improved (Group 1), stable (Group 2), and worsened (Group 3) cognition as defined by the Alzheimer’s Disease Assessment Scale-Cognitive 13 score change from baseline to 16 weeks. CSF indicates cerebrospinal fluid. Data are mean ± standard error mean (SEM).
